# Robotic resection of cardiac papillary fibroelastoma guided by three-dimensional transesophageal echocardiography: a case report

**DOI:** 10.31744/einstein_journal/2026RC1564

**Published:** 2026-10-13

**Authors:** Fernando Hideki Sakamoto, Ana Clara Tude Rodrigues, Claudio Henrique Fischer, Edgar Lira-Filho, Samira Saady Morhy, Adriana Venturini Reche, Robinson Poffo, Sergio Augusto Fudaba Curcio, João de Azevedo, Sofia Alves Figueiredo Faustino, José Henrique Dias de Sousa, Marcelo Luiz Campos Vieira

**Affiliations:** 1 Hospital Israelita Albert Einstein São Paulo SP Brazil Hospital Israelita Albert Einstein, São Paulo, SP, Brazil.

**Keywords:** Heart neoplasms, Cardiac papillary fibroelastoma, Echocardiography, transesophageal, Mitral valve, Robotic surgical procedures

## Abstract

Heart neoplasms are classified as primary or secondary, with primary tumors considered rare. Cardiac papillary fibroelastoma is the second most common benign cardiac tumor. It has distinct morphological features and carries a potential risk of embolization; therefore, surgical excision is generally recommended. The use of three-dimensional transesophageal echocardiography (3D TEE) enables improved characterization of the suspected lesion, provides a detailed anatomical assessment, and assists in intraoperative planning and management. We report the case of a patient diagnosed with a cardiac papillary fibroelastoma of the mitral valve who underwentrobotic-assisted surgery guided by 3D TEE.

## INTRODUCTION

Although rare, heart neoplasms comprise two major groups that require appropriate investigation and management.^(1)^ Primary heart neoplasms are benign in up to 90% of cases and malignant in approximately 10%.^(2,3)^ Among benign tumors, cardiac papillary fibroelastoma is the second most common type. The diagnosis of these lesions requires a high index of clinical suspicion, as they exhibit distinct morphological features. Three-dimensional transesophageal echocardiography (3D TEE) provides superior anatomical and morphological characterization, facilitating accurate diagnosis and guiding clinical decision-making, particularly given the significant risk of embolization and associated morbidity.^(4,5)^

## CASE REPORT

We present the case of a 48-year-old male patient with a history of chondrosarcoma of the right shoulder and no prior cardiovascular or cerebrovascular disease. He had episodes of transient ischemic attack (TIA) in 2013 and 2018. Following those events, investigations including cranial computed tomography, brain magnetic resonance imaging, carotid Doppler ultrasonography, 24-hour Holter monitoring, and transthoracic echocardiography were performed, but no cardioembolic or atherothrombotic sources or arrhythmias were identified. Thrombophilia testing was also negative.

During follow-up, the patient remained asymptomatic. In November 2024, a routine transthoracic echocardiogram (TTE) revealed a newly identified oval mass on the atrial surface of the mitral valve. For better characterization, two-dimensional (2D) and three-dimensional (3D) transesophageal echocardiography (TEE) were performed, confirming an oval, pedunculated, measuring 8mm, mobile structure and adherent to the atrial surface of the anterior mitral leaflet (segment A1), suggestive of a cardiac papillary fibroelastoma (CPF). The remainder of the mitral valve complex appeared normal. During the examination, after injection of agitated saline, a small number of bubbles were visualized crossing from the right atrium to the left atrium through the fossa ovalis membrane between the third and the fourth heartbeat, raising suspicion of a patent foramen ovale (PFO).

As part of the preoperative assessment, non-invasive coronary evaluation was performed, showing no evidence of myocardial ischemia. Repeated 24-hour Holter monitoring and carotid Doppler ultrasonography were also normal. Subsequently, after all examinations were completed, the patient underwent robotic-assisted thoracoscopic surgery (RATS) for excision of the mitral valve lesion.

The procedure took place in November 2024. After left atriotomy and exposure of the mitral valve, a pedunculated lesion adherent to the atrial surface of the anterior mitral leaflet (segment A1) was confirmed (Figure 1), followed by *en bloc* resection. Postoperatively, the mitral valve remained competent (Figure 1), and no evidence of a PFO was observed intraoperatively.

**Figure 1:**
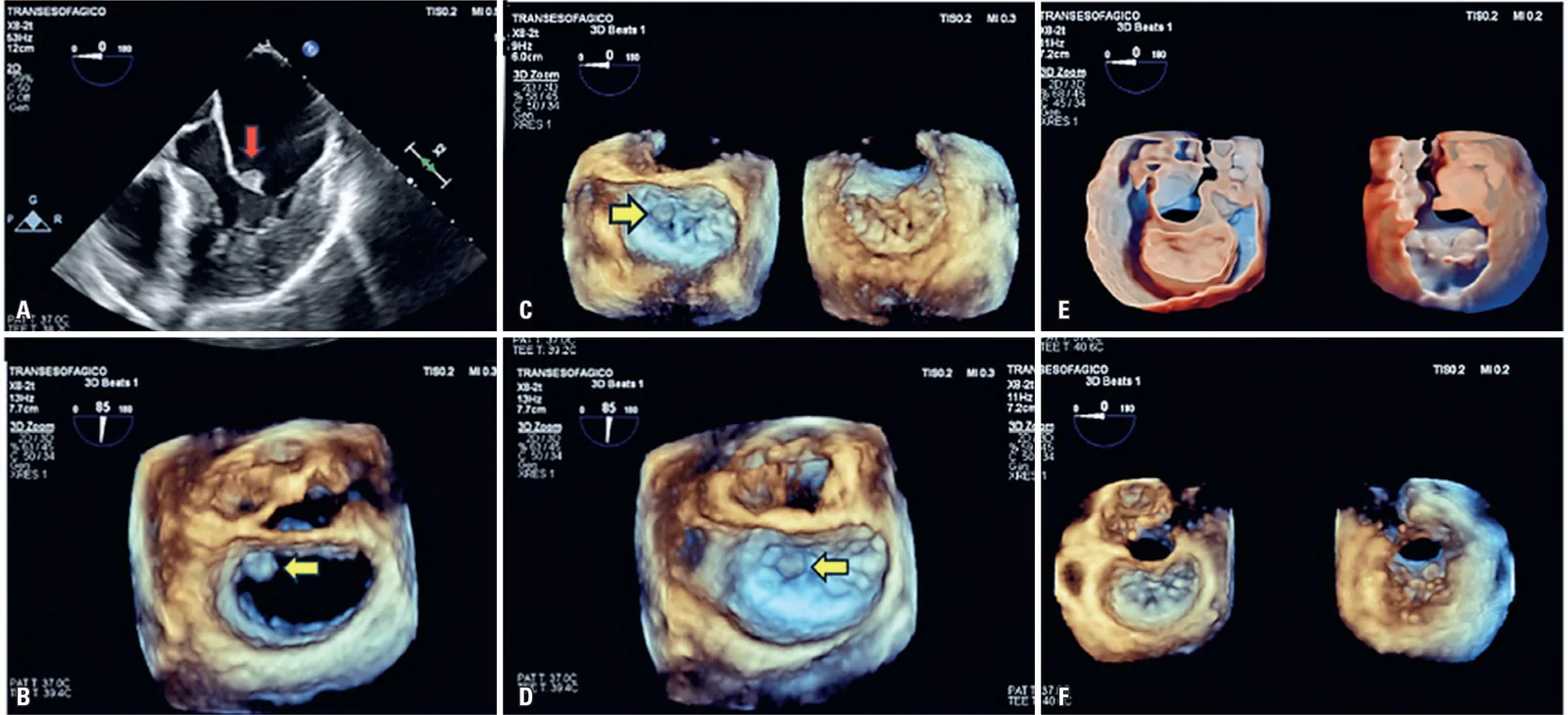
(A) Mid-esophageal 2D TEE view at 0°, showing an oval lesion (red arrow) adherent to the A1 segment of the anterior mitral leaflet; (B) mid-esophageal 3D TEE view at 85° showing an oval lesion (yellow arrow) adherent to the A1 segment of the anterior mitral leaflet, with the valve open; (C) mid-esophageal 3D TEE view at 0°, showing two perspectives: an atrial view on the left (yellow arrow indicating the lesion) and, a ventricular view on the right; (D) mid-esophageal 3D TEE view at 85°, showing an oval lesion (yellow arrow) adherent to the A1 segment of the anterior mitral leaflet, with the valve closed; (E) mid-esophageal 3D TEE TrueVue Glass view at 0° showing the mitral valve after excision of the lesion; (F) mid-esophageal 3D TEE view at 0°, showing the mitral valve after excision of the lesion

The patient had an uneventful recovery and was discharged on postoperative day four. Histopathological examination (Figure 2) confirmed the diagnosis of CPF. Follow-up 2D TTE (Figure 2) demonstrated a mitral valve with minimal regurgitation, preserved leaflet motion, and no signs of tumor recurrence. The patient remained free of cerebrovascular events.

**Figure 2:**
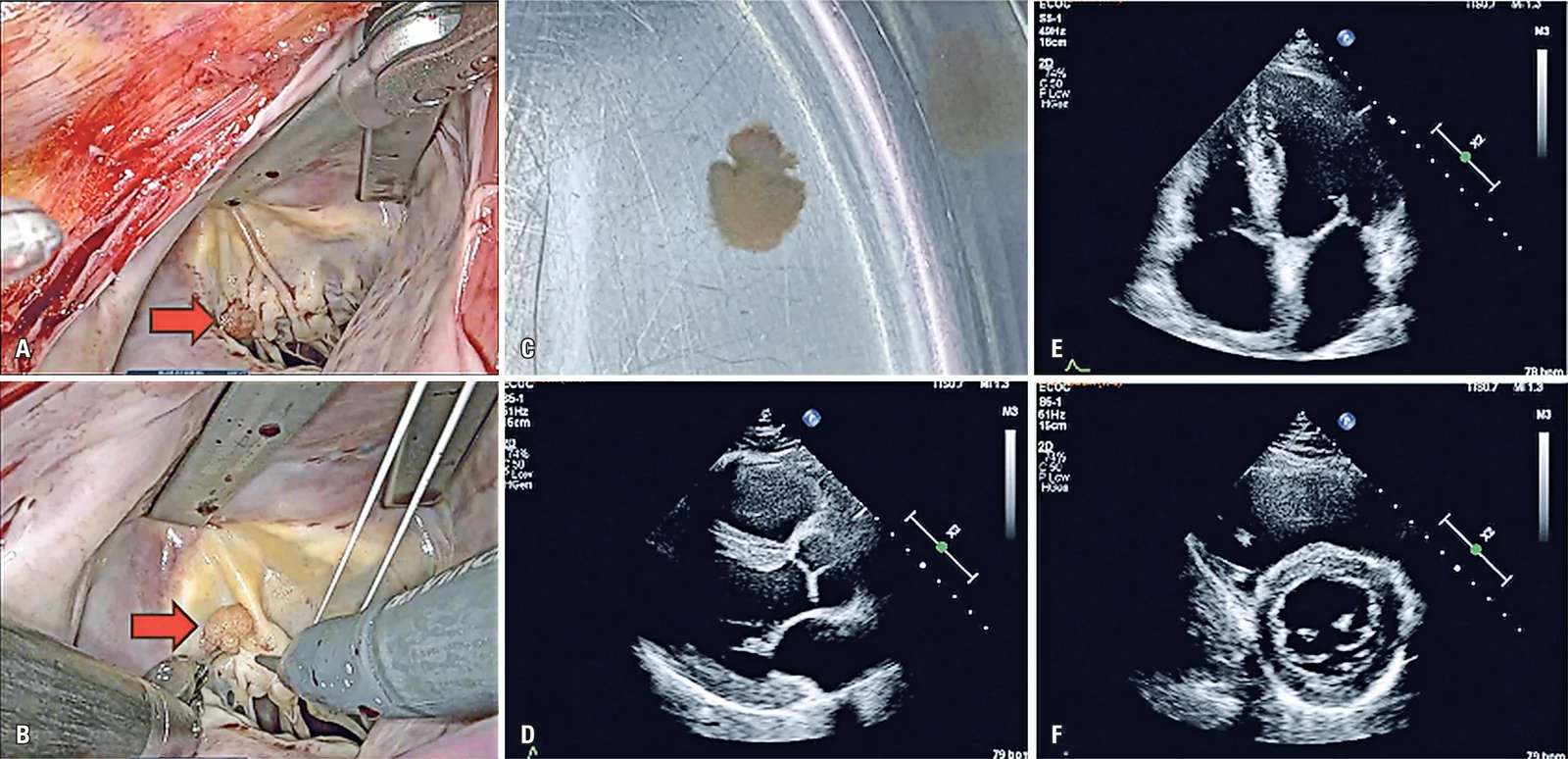
(A and B) Intraoperative robotic images showing a fixed oval lesion with a short stalk adherent to the atrial surface of the A1 segment of the anterior mitral leaflet (red arrow); (C) excised lesion with a sea anemone appearance; (D, E and F) postoperative transthoracic echocardiographic images showing no significant valvular abnormalities or lesion recurrence

This article was approved by the ethics committee of Hospital *Israelita Albert Einstein* (CAAE: 85306624. 2.0000.0071; # 7.279.192).

## DISCUSSION

Heart neoplasms are classified into two groups: primary and secondary cardiac tumors.^(1,2)^ Primary heart neoplasms are significantly less prevalent than secondary tumors and are considered rare entities, with a reported prevalence ranging from 0.001% to 0.03%.^(1,2)^ Their prevalence increases with age. Benign primary heart neoplasms exhibit distinct characteristics,^(3)^ with the most common types being myxomas (the most prevalent in most studies), rhabdomyomas, lipomas, hemangiomas, and cardiac papillary fibroelastomas (CPFs). According to recent studies, CPF is the second most common benign primary heart neoplasm.^(1–3)^

There is no definitive consensus on the pathogenesis of these lesions, although associations have been proposed with prior viral infections and mutations in the Kirsten rat sarcoma (KRAS) oncogene. Kirsten rat sarcoma mutations may contribute to progressive tumor growth, with diagnosis becoming more frequent after the sixth decade of life.^(4)^

Cardiac papillary fibroelastomas can originate from any cardiac structure, although they are most commonly found on the heart valves (in more than 80% of cases). Their distribution, in decreasing order of frequency, is as follows: the aortic, mitral, tricuspid, and pulmonary valves. They typically appear as solitary, oval-shaped lesions with a short, fixed stalk and well-defined borders. A key differential diagnosis not classified as a tumor is Lambl's excrescences (LEs), which are filiform, mobile structures that are often multiple and typically located at valvular coaptation lines. Although CPFs and LEs may share similar histological features, they can often be distinguished by imaging. CPFs are usually solitary, oval, less mobile, and less filiform than LEs.^(4,5)^ Therefore, 2D and 3D TEE are particularly useful in differentiating CPFs from malignant neoplasms, vegetations associated with infective endocarditis, LEs, and thrombi.

Histopathologically, CPFs are composed of avascular papillary fronds lined by endothelial cells, often containing adherent fibrin and thrombi ^(3,4,5)^ which confer embolic potential. Systemic embolic events, such as stroke and transient ischemic attack (TIA), can occur. This embolic risk, combined with the progressive growth of the lesion,^(2,3,5)^ may explain why previous echocardiographic evaluations failed to detect the lesion, which only became evident on more recent 2D TTE examination and was subsequently confirmed by 2D and 3D TEE.

In this case report, after identification of the mitral valve lesion, robot-assisted thoracoscopic surgery (RATS) was selected as the surgical approach. Although RATS is widely used in North America and Europe, data on its application in Brazil and Latin America remain limited.^(6,7)^ The procedure was performed at one of the largest robotic surgery centers in Brazil, which offers advanced echocardiographic resources, an experienced surgical team, and low complication rates potentially contributing to a shorter hospital stay.^(7)^

Since the introduction of echocardiography, 2D and 3D imaging techniques have evolved significantly. Advances such as the miniaturization of ultrasound components, the development of more ergonomic transducers, and the introduction of matrix-array technology have expanded the clinical and surgical applications of 3D TEE.^(8,9)^ The latest developments — such as TrueVue and TrueVue Glass imaging — enable enhanced surgical planning, highly realistic anatomical visualization, intraoperative precision, real-time assessment of cardiac structures, and a reduction in artifacts and angle-dependent limitations. These innovations make 3D TEE an invaluable tool in cardiac surgery, particularly in the context of robotic techniques.^(9,10)^

In this case, 2D and 3D TEE played a crucial role in lesion characterization, exclusion of thrombi and vegetations, localization of the implantation site, evaluation of potential structural damage to the mitral apparatus, and confirmation of the absence of acute postoperative complications. Thus, these imaging modalities proved to be essential at all stages, from diagnosis to surgical planning and postoperative management.

## CONCLUSION

The diagnostic confirmation of cardiac papillary fibroelastoma has become increasingly frequent with advances in imaging techniques such as 2D and 3D echocardiography, particularly with the use of TrueVue and TrueVue Glass imaging. These technologies provide greater diagnostic accuracy, improved anatomical correlation with intraoperative findings, and support for postoperative follow-up. This report therefore offers an updated perspective on the diagnosis, management, and follow-up of cardiac papillary fibroelastoma.

## Data Availability

All data supporting the findings of this case report are included in the manuscript.
